# HEV-Associated Neuralgic Amyotrophy: A Multicentric Case Series

**DOI:** 10.3390/pathogens10060672

**Published:** 2021-05-30

**Authors:** Johannes H. Bannasch, Benjamin Berger, Claus-Peter Schwartkop, Marco Berning, Oliver Goetze, Marcus Panning, Miriam Fritz-Weltin, George Trendelenburg, Mathias Gelderblom, Marc Lütgehetmann, Fridrike Stute, Thomas Horvatits, Meike Dirks, Christoph Antoni, Patrick Behrendt, Sven Pischke

**Affiliations:** 1 Institute of Medical Microbiology, Virology and Hygiene, University Medical Centre Hamburg-Eppendorf, 20251 Hamburg, Germany; j.bannasch@gmx.de (J.H.B.); mluetgehetmann@uke.de (M.L.); 2 Department of Medicine, University Medical Centre Hamburg-Eppendorf, 20251 Hamburg, Germany; t.horvatits@uke.de; 3 German Center for Infection Research (DZIF), Hamburg-Lübeck-Borstel and Heidelberg Partner Sites, 20359 Hamburg, Germany; 4 Clinic of Neurology and Neurophysiology, Medical Centre—University of Freiburg, Faculty of Medicine, University of Freiburg, 79106 Freiburg, Germany; benjamin.berger@uniklinik-freiburg.de (B.B.); miriam.fritz@uniklinik-freiburg.de (M.F.-W.); 5 Clinic for Neurology, Medical Centre Itzehoe, 25524 Itzehoe, Germany; c.schwartkop@kh-itzehoe.de (C.-P.S.); g.trendelenburg@kh-itzehoe.de (G.T.); 6 Department of Medicine I, University Medical Center Dresden, Technische Universität (TU) Dresden, 01307 Dresden, Germany; marco.berning@uniklinikum-dresden.de; 7 Division of Hepatology, Department of Medicine II, University Hospital Wurzburg, 97080 Würzburg, Germany; goetze_o@ukw.de; 8 Institute of Virology, Medical Center—University of Freiburg, Faculty of Medicine, University of Freiburg, 79104 Freiburg, Germany; marcus.panning@uniklinik-freiburg.de; 9 Department of Neurology, University Medical Centre Göttingen, 37075 Göttingen, Germany; 10 Clinic for Neurology, University Medical Centre Hamburg-Eppendorf, 20251 Hamburg, Germany; m.gelderblom@uke.de; 11 Clinic for Pediatrics, University Medical Centre Hamburg-Eppendorf, 20251 Hamburg, Germany; f.stute@uke.de; 12 Clinic for Neurology and Neurophysiology, Medical School Hannover, 30625 Hannover, Germany; dirks.meike@mh-hannover.de; 13 Department of Medicine II, University Medical Center Mannheim, Medical Faculty Mannheim, Heidelberg University, 68167 Mannheim, Germany; christoph.antoni@umm.de; 14 Department for Gastroenterology, Hepatology und Endocrinology, Medical School Hannover, 30625 Hannover, Germany; Behrendt.Patrick@mh-hannover.de

**Keywords:** Hepatitis E, HEV, neuralgic amyotrophy, NA

## Abstract

Background: Neuralgic amyotrophy (NA) has been described as a possible extrahepatic manifestation of hepatitis E virus (HEV) infection. Usually, HEV-associated NA occurs bilaterally. The clinical characteristics determining the course of HEV-associated NA have still not been defined. Methods: In this retrospective multicentric case series, 16 patients with HEV-associated NA were studied and compared to 176 HEV patients without NA in terms of their age, sex, and ALT levels. Results: Neither gender distribution (75% vs. 67% male) nor age (47 vs. 48 years median) differed significantly between the NA patients and controls. Eight NA patients (50%) presented with bilateral involvement—seven of these had right-side dominance and one had left-side dominance. Thirteen cases (81%) were hospitalized. Eight of these patients stayed in hospital for five to seven days, and five patients stayed for up to two weeks. The time from the onset of NA to the HEV diagnosis, as well as the diagnostic and therapeutic proceedings, showed a large variability. In total, 13 (81%) patients received treatment: 1/13 (8%) received intravenous immunoglobulins, 8/13 (62%) received glucocorticoids, 3/13 (23%) received ribavirin, and 6/13 (46%) received pregabalin/gabapentin. Patients with ages above the median (47 years) were more likely to be treated (*p* = 0.001). Conclusion: HEV-associated NA causes a relevant morbidity. In our case series neither the type of treatment nor the time of initiation of therapy had a significant effect on the duration of hospitalization or the course of the disease. The clinical presentation, the common diagnostic and therapeutic procedures, and the patients’ characteristics showed large variability, demonstrating the necessity of standardized protocols for this rare but relevant disease.

## 1. Introduction

Hepatitis E virus (HEV) infections present diverse diseases. For more than three decades, Hepatitis E, which causes inflammation of the liver, has been the predominant clinical presentation of this disease, but in recent years, awareness of possible extrahepatic manifestations has emerged [1]. Various neurological diseases, particularly neuralgic amyotrophy (NA), have been observed in the context of acute or chronic HEV infections, but proof of causality for the majority of these assumed HEV-induced diseases, including NA, is still pending [1]. Thus, more clinical data and pathophysiological insights are needed to characterize and understand this assumed relationship.

NA (also called Parsonage turner syndrome or brachial neuritis) describes a post- or parainfectious inflammation of the brachial plexus. This rare disease occurs in approximately 2–4 out of every 100,000 people per year. The clinical picture is characterized by severe neuropathic shoulder and arm pain with an acute onset, often culminating in multifocal muscle paresis and atrophy over the further course of development. Involvement of nerves from other regions has also been described in 23% of cases [2]. The pathogenesis of NA has not yet been clarified, but a multifactorial genesis is assumed [3,4]. Some studies and case reports suggest that HEV is a possible causal factor for NA [1,5,6].

A study published in 2014 with 47 NA patients from the United Kingdom (UK) and the Netherlands showed that 5/47 (11%) cases had acute HEV infections [2]. Patients presented with almost normal bilirubin and ALT levels. Positive anti-HEV IgM antibodies were not associated with age, gender, severity, or outcome of the disease. Based on these findings, another multicenter retrospective study with 57 HEV-positive NA patients (HEV-NA) and 61 HEV-negative controls with NA was carried out by Van Eijk et al. [6]. This study revealed that the majority of HEV-positive patients were male and middle-aged (mean age: 51 years), and thus slightly older than the HEV-negative patients (mean age: 44 years). The HEV-NA patients had bilateral involvement of the brachial plexus significantly more frequently than the HEV-negative patients (HEV-positive, 80.0% vs. HEV-negative, 9%). In addition, the HEV-NA cohort suffered significantly more frequently from further neurological abnormalities outside the brachial plexus, including involvement of the phrenic nerve and the lumbosacral plexus, as well as decreased tendon reflexes in the affected limbs [6].

In addition to these findings, a multicentric study by Dalton et al. prospectively studied a large cohort of 464 non-traumatic neurological patients for the presence of HEV with polymerase chain reaction (PCR) [7]. Three out of the five NA cases in this study were associated with HEV.

Furthermore, Ripellino et al. observed a strong association between NA and HEV, especially with the development of a distinct clinical phenotype [8,9]. In a prospective Swiss study, 141 acutely HEV-infected patients were examined for neurological symptoms; 43 (31%) showed abnormalities [9]. In 15/141 HEV-infected patients (11%), the symptoms corresponded to clinically diagnosable NA. This study also showed a clinically dominant phenotype with bilateral, albeit asymmetrical, involvement of the brachial plexus (10/15, 67%). Antibodies against gangliosides were not present in any of the HEV-NA patients (0/15) [8].

In summary, NA has been linked to HEV infections in previous reports, but the clinical characteristics that determine the development of HEV-associated NA and the clinical course are still little known. Thus, the present study aimed to analyze the clinical characteristics of a multicentric German case series containing 16 patients with HEV-associated NA and to identify factors associated with the course of the disease.

## 2. Patients and Methods

### 2.1. Patients

In this retrospective multicentric analysis, the clinical characteristics of all known cases of HEV-associated NA (n = 16) at the University Hospital Hamburg-Eppendorf, University Hospital Freiburg, University Hospital Mannheim, University Hospital Carl Gustav Carus Dresden, Hannover Medical School, Klinikum Itzehoe, and University Hospital Würzburg were analyzed through a chart review. The parameters assessed included the age, sex, pre-existing conditions, date of symptom onset of neuralgic amyotrophy, date of HEV testing, affected side, HEV serology, HEV PCR in blood and cerebrospinal fluid (CSF), maximum value of ALT, affected muscles, time period in days until the NA complaints normalize, diagnostic tests, imaging procedures, therapy, and hospitalization period in days.

The diagnosis of NA is exclusively clinical and occurs after ruling out other causes. Differential diagnoses to be excluded are: inflammation in the cerebrospinal fluid (CSF), trauma, and nerve compression of the cervical spine and brachial plexus. All patients in this study were previously diagnosed with NA, as well as HEV infection, and then these patients were identified through a retrospective database search.

A cohort of 176 patients with HEV infections and without NA served as controls (28 immunosuppressed patients with chronic HEV infections, 143 immunocompetent patients with acute Hepatitis E, and 5 blood donors with asymptomatic HEV infections). This retrospectively studied cohort included HEV-positive patients who presented at the University Hospital Hamburg Eppendorf from March 2011 to October 2018.

### 2.2. Methods

For serum analysis, the following commercially available ELISA (enzyme-linked immunosorbent assay) or CLIA (chemiluminescent-linked immunosorbent assay) kits were used: Mikrogen, Neuried, Germany (n = 9); Wantai, Beijing, PR China, (n = 3); Vircell, Granada, Spain (n = 2); and other (n = 2). PCR testing was performed with a Roche Cobas Taqman 6800 (n = 5), Altona diagnostics Realstar HEV RT-PCR kit (n = 6), and various other PCR assays (n = 5) according to the manufacturers’ instructions (Roche, Hilden, Germany, as well as Altona Diagnostics, Hamburg, Germany).

### 2.3. Definition of Hepatitis E Virus Infection

Acute HEV infection was diagnosed by either detecting HEV RNA in the serum using PCR or with reactive anti-HEV IgM in combination with the typical clinical course of elevated transaminases.

### 2.4. Statistics

Continuous variables are described as the median and standard deviation. For categorical variables, absolute and relative numbers are presented. Continuous variables were compared using the Mann–Whitney U test and categorical variables were compared using chi-square tests. Statistical analysis was performed with SPSS version 13. *p*-values less than 0.05 were regarded to be statistically significant.

### 2.5. Ethics

The study was conducted according to the guidelines of the Declaration of Helsinki. Data collection at the University Hospital Hamburg Eppendorf was approved by the Ethics Committee of the Medical Council of Hamburg (WF-138/20 and PV7049). Data from other centers were transmitted anonymously to the Hamburg center and were analyzed in line with the local recommendations of all other hospitals and ethics committees involved.

It was not possible to get written informed consent from the patients in this anonymously blinded cohort. Cases have been recruited at several German centers anonymously analyzed. This proceeding is in line with the ethical courts of the participating centers and this proceeding is usual for retrospective anonymous cohorts.

## 3. Results

### 3.1. HEV Cohort with Neuralgic Amyotrophy

Sixteen patients with neuralgic amyotrophy (NA) were included in this study. Twelve individuals were male (75%). Fifteen patients (94%) suffered from acute infection, and one patient from chronic HEV infection (viremia > 6 months; Case #1). The median age at NA onset was 47 years (range: 26–57 years). Anti-HEV IgM antibodies were present in 15 patients (94%). In 7/15 patients, the additional HEV PCR test was positive (Table 1). In one serological negative patient (6%), the diagnosis of HEV infection relied solely on the positive PCR result. Cerebrospinal fluid (CSF) specimens were available from seven patients. Only two of these seven CSF samples (29%) tested positive for HEV through PCR. Half of the patients had no relevant previous medical conditions (n = 8). The remaining patients suffered from cardiovascular diseases (n = 3), herniated discs (n = 2), bronchial asthma (n = 1), heart transplantation combined with chronic HEV infection (n = 1), and multiple sclerosis (n = 1).

A total of 50% of the patients had a bilateral distribution pattern (Table 2). In unilateral appearance, the right side clearly prevailed in 7/8 of these (88%). In 13 patients, the symptoms were initially a combination of severe neuropathic pain and hypesthesia. In the other three cases, neuropathic pain without senso-motoric symptoms was reported. All patients showed a classical effect on the shoulder–arm region. The main affected muscles were: M. serratus anterior (n = 12), M. deltoideus (n = 11), Mm. supraspinatus (n = 9), and infraspinatus (n = 9). Additionally, four patients (25%) suffered from extrabrachial manifestations: diaphragm muscle (n = 2), M. quadriceps (n = 2), M. gluteus maximus (n = 1), and nervus trigeminus (n = 1).

**Figure 1:**
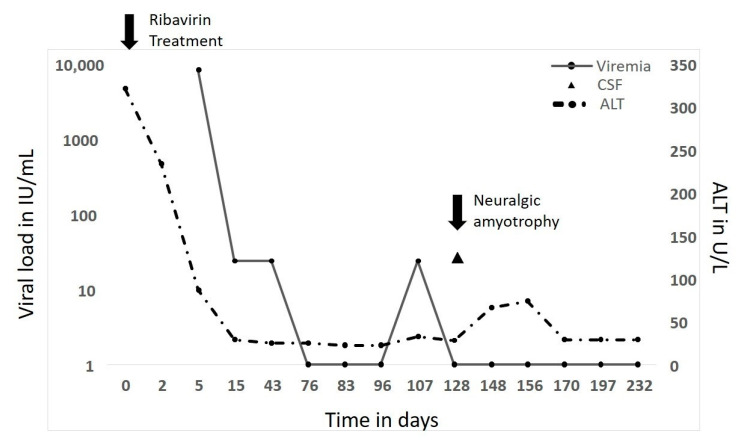
Course of ALT and HEV viral load in a chronically infected patient (Patient #1) that developed NA under treatment with ribavirin.

The time from the onset of symptoms of NA to diagnosis showed a large variability (Table 2). Only one patient (Patient #1, heart transplant recipient) was suffering from chronic HEV infection and developed NA under ongoing ribavirin treatment (Figure 1). Another patient was a woman with multiple sclerosis suffering from various neurological symptoms, which have initially been associated with her multiple sclerosis. During the further course she developed slightly elevated liver enzymes and tested positive for HEV by PCR. To prevent acute worsening of her condition a ribavirin therapy has been initiated. During the further course the clinical picture changed and a typical NA has been diagnosed. However, as the first presentation was outside of any of our centers, we cannot definitively state if the first presentation of neurological symptoms has been falsely classified as multiple sclerosis associated symptoms, thus we cannot depict more details of this particular case. All other patients have been diagnosed to suffer from HEV infection simultaneously or directly after presentation at the participating centers. However, at this time point some patients were already suffering from NA for multiple weeks. Some of these protracted only tested positive for anti HEV IgM and IgG but already negative by PCR.

In three patients, the HEV genotype could be determined; all suffered from Genotype 3, the endemic HEV genotype in Europe. In eight patients, the IgG total was determined, which was normal (median: 10.2, range: 7.0–14.6 g/L) in all of them. Four patients were tested for presence of cryoglobulins during the episode of NA; none tested positive.

### 3.2. Diagnostic and Imaging Procedures

Various diagnostic tests were used to assess the neurological injuries: electromyography (EMG) (n = 12), nerve conduction studies (NCSs) (n = 11), spinal magnetic resonance imaging (MRI) (n = 10), and sonography (n = 2). The results of the electrophysiological tests (EMG and NCS) presented the most variable images. In 7/12 (58%) of the cases, EMG showed chronic neurogenic damage; in 5/12 cases (42%), it showed spontaneous muscle activity as a sign of fluoride denervation, and in 2/12 (17%) patient, normal findings were observed. In 5/11 (46%) cases, NCSs demonstrated signs of axonal injury, while 5/11 (46%) cases also showed normal results, and in one case (8%), no nerve response could be detected by the NCSs.

While characteristics associated with NA, such as swelling of the plexus, could be found in five patients by MRI, in two patients, ultrasounds were performed as an alternative to MRI, which showed proof of nerve swelling.

### 3.3. Individual Therapeutic Approaches

In 13 cases, various immunotherapeutic and/or symptomatic drugs were used. Specific therapy was given to eight of these: 1/13 (8%) intravenous immunoglobulins, 8/13 (62%) glucocorticoids, and 3/13 (23%) ribavirin (Table 3). In all specifically treated patients (n = 8) this treatment has been initiated within 14 days. Non-specific pain medication was given to 6/13 (46%) with pregabalin/gabapentin. In three of these cases, several drugs were combined simultaneously. In one patient with chronic HEV infection (Case #1), ribavirin was started more than three months prior to the onset of NA; in the remaining five ribavirin-treated patients, treatment was initiated due to severe HEV-associated NA. The patients that received a specific therapy had a significant higher age in comparison to the patients that did not receive specific therapy (*p* = 0.001). In 13 cases, hospitalization was necessary. Patients with an age below the median of 47 years tended to be hospitalized for shorter periods in comparison to older patients. However, this failed to reach significance (*p* = 0.06). Eight patients stayed in the hospital for 5 to 7 days, and five patients even spent up to 2 weeks (Table 4).

### 3.4. Case Reports of Particularly Interesting Cases

Two patients presented uncommon and unique cases of HEV-associated NA with particular novel features.

- Case #1

A 46-year-old male received a heart transplant at the age of 40 for dilatative cardiomyopathy. To prevent rejection, he was given permanent immunosuppressive medication consisting of everolimus (through level 5–10 µ/L), mycophenolate mofetil (360 mg twice daily), and prednisolone (5 mg daily). Seven years after transplantation, chemically elevated liver parameters were observed in a laboratory and a chronic Hepatitis E infection with a high viral load (6 million IU/mL) was diagnosed. Under ribavirin treatment, the liver parameters and viral load normalized, but the patient developed NA four months after the initiation of prolonged ribavirin therapy (Figure 1). A short blip of the HEV viral load (PCR) immediately preceded the onset of NA. HEV could be detected in his cerebrospinal fluid at a low concentration (PCR). No additional therapies (intravenous immunoglobulin (IVIG), steroid dosage increase) were used in this patient. Over 6 months after the onset of NA symptoms, they completely vanished.

- Case #2

A 48-year-old female patient presented in the emergency room with acute pain in the shoulder and weakness in the arms. With her pre-existing conditions, she mentioned a vertebral body operation in the lumbar spine area and a car accident a year before, thus she was treated by a neurosurgeon and has not been seen by a neurologist. An MRI scan showed a slipped disc cervical vertebrae 5/6, which was classified as the most probable cause. NA has not been suspected and an EMG has not been performed. However, neurosurgical intervention did not improve the paresis. Due to increased liver values, the patient was tested for HEV, resulting in a positive blood HEV PCR test, a positive anti-HEV IgM test, and borderline IgG titers, which finally led to the diagnosis of NA and acute hepatitis E within one week after operation.

### 3.5. Comparison with a Control Group of HEV-Infected Patients without NA

The control group of HEV-infected patients without NA (n = 176) did not differ significantly from the NA group in terms of the gender distribution, ALT levels, or age (117 men, 67%; ALT: 19–6790 IU/mL, mean 870 IU/mL, STD 1267 IU/mL; age: 18–81 years, median 48 years, STD 15 years; no statistical differences according to chi-square or Mann–Whitney testing).

## 4. Discussion

The comparison of the HEV-NA patients with a cohort of HEV-infected patients without NA did not allow us to identify predictors for the development of HEV-NA, as the sexes, ages, and ALT levels did not differ significantly between these groups.

The present case series highlights the severity and morbidity of HEV-NA, which resulted in 81% (13/16) of cases requiring hospitalization. Particularly, in patients with an age above the median of 47 years, the disease caused a long course. Various medicinal treatments were tried, particularly in the older patients, but in this case series, none of these drugs (ribavirin, steroids, IVIG, and combinations of these) were shown to be superior. Neither treatment form nor timing resulted in a significantly different time of hospitalization or milder/shorter disease course. Furthermore, the patients receiving the various therapy types in this retrospective analysis were not comparable, as there was no standardized treatment algorithm. As shown in Table 3, patients with an age above the median of 47 years received specific treatments (steroids, IVIG, ribavirin) significantly (*p* = 0.001) more frequently than to younger patients did. However, it remains unclear if this phenomenon was caused by a more severe course in the elderly or if the treating physicians more frequently decided to initiate specific treatments in elderly patients in the face of more underlying comorbidities. Prospective multicentric cohorts are needed to clarify this aspect.

In addition to the treatment options, the diagnostic proceedings showed a huge variability. While the majority of HEV-NA patients in the present cohort underwent electromyography (n = 12/16), nerve conduction studies (NCSs) (n = 11/16), and magnetic resonance imaging (MRI) (n = 10/16), only a minority received ultrasound examinations of the brachial plexus (n = 2/16). Thus, international guidelines as well as standardized diagnostic and treatment algorithms are mandatory, while single national associations, such as the German Association of Neurologists, have already mentioned testing for HEV in NA patients in their guidelines (https://dgn.org/leitlinien/ll-030-067-diagnostik-bei-polyneuropathien-2019/ accessed on first of January 2021). These specifications would help to improve the understanding of this rare disease and the care of the patients. Particularly, ultrasound presents a cheap and non-invasive technique and can easily be performed in the hands of an experienced investigator, as described previously [10,11,12].

A particular case that illuminated the possible consequences of misleading treatment algorithms was described in Case #2: the pre-existing conditions, the symptoms, and the MRI unfortunately resulted in the decision of the neurosurgeon to assume that a herniated disc was the cause and to treat this through an operative spinal fusion. This cases clearly demonstrates that such patients should be presented to neurologists which might help to avoid false diagnosis in this context and recognize the rare NA, particularly the HEV associated NA.

In the present study, the male patients suffered more frequently than female patients from HEV-associated NA (75%). In comparison to the control cohort (67% male), this failed to reach statistical significance (*p* = 0.56); thus, the unbalanced gender distribution in the NA group should not be overestimated. In line with this finding, a systematic review of multiple published cases of HEV-associated NA demonstrated that 87% of HEV-associated cases of NA (34/39) were male in comparison to the 68% (136/199) of general NA patients that were male (*p* < 0.02) [13]. Indeed, the present study confirms that men suffer from HEV-associated NA more frequently than women, but given the control cohort, this could be identified as a feature of HEV infections in general, and not as specific sign of HEV-associated NA. The different food consumption habits, particularly consumption of undercooked swine meat, in men and women probably result in a higher risk of HEV exposure. Furthermore, genetic and immunological sex differences might influence the probability of developing NA after exposure to HEV.

A particular case observed in this series was the case of Patient #1 (heart transplant recipient): he had been chronically infected with HEV for more than two years when he developed NA. Two interesting findings can be observed in this particular case: (i) he developed NA despite ongoing ribavirin treatment, and (ii) a sudden blip in the viral load preceded the development of NA (Figure 1). Thus, it can be concluded that ribavirin does not protect against the development of NA, but the development of NA may be caused by neuropathic strains, which were causative for this blip. Unfortunately, this hypothesis could not be proven, as the viral load was too low to compare the strain from the patient’s CSF with the strains from his serum. Recently, a case of chronic HEV infection of the central nervous system leading to hyperesthesia of the arms and legs and motor dysfunctions without paresis was described in an immunocompetent man [14]. Under ribavirin therapy, detectable HEV RNA dissolved in the serum and stool, while persistent HEV infection could be repeatedly detected in the CSF for more than three years. Sanger sequencing revealed a four-amino-acid in-frame deletion and an additional eight-amino-acid in-frame deletion in comparison to a reference HEV strain (KX172133).

Recently, it has been demonstrated that HEV strains in the ejaculate of chronically HEV infected patients differ genetically from strains in the blood of these patients demonstrating replication beyond the blood-testis barrier [15]. Similarly, the evidence of neuropathic strains and specific variants resulting in the development of NA can be hypothesized.

In contrast to the hereby depicted patient with a distinct neurological clinical picture, only 2/7 (29%) available CSF samples tested positive for HEV via PCR, suggesting a different pathomechanism from the ongoing inflammation of HEV-infected neuronal tissue. Perhaps the damage of neuronal tissue and the slow regeneration time of neuronal tissue are the explanation for the long-lasting symptoms of NA in numerous patients. Half of the patients in our cohort had ongoing symptoms for more than three months. In line with these findings, long time persistence of NA-associated symptoms has been reported previously [16,17,18].

Despite the present novel insights into the clinical picture of HEV-associated NA, however, the retrospective study design causes various limitations founded by the various diagnostic and therapeutic procedures at the various participating centers. Furthermore, due to our small number of patients, statistical analyses are not reliable. In addition to the medicinal treatment, particularly the neurological examination of the involved muscles and nerves requires a standardized procedure and should be evaluated prospectively in further studies.

In conclusion, the variability of NA diagnosis and treatment, as well as the various clinical proceedings, may result one the one hand in a prolonged time until an underlying HEV infection is found and, possibly, in the various outcomes of the disease. In 4/16 patients, it took, on average, more than 18 weeks to establish the correlation between NA and HEV. On the other hand, the above-mentioned variability makes it difficult to identify which medicinal treatment may shorten or attenuate the clinical course. Therefore, international guidelines and strict recommendations for neurological professional societies are needed in order to standardize these topics.

## Figures and Tables

**Table 1:** Diagnostic procedure in Hepatitis E virus (HEV) testing.

	Diagnosis Based Solely on Positive Serology (8 Patients)	Diagnosis Based Initially on Positive PCR (8 Patients)
Anti-HEV positivity		
-IgG	7/8	7/8
-IgM	8/8	7/8
Age	45 years (32–50)	46 years (26–57)
Sex	7× male	5× male
	1× female	3× female
Symptoms	7× pain + hypesthesia	6× pain + hypesthesia
	1× pain solely	2× pain solely
Time to HEV diagnosis	51 days (1–270) *	30 days (0–180) *
Affected side	4/8 unilateral right	3/8 unilateral right
		1/8 unilateral left
	4/8 bilateral	4/8 bilateral
ALT (U/L)	870 (71–2366)	611 (22–1331)
Affected muscles	7/8 M. serratus anterior	5/8 M. serratus anterior
		4/8 M. deltoideus
	7/8 M. deltoideus	3/8 M. supraspinatus
	6/8 M supraspinatus	3/7 M. infraspinatus
	6/8 M. infraspinatus	1/8 Diaphragm
	1/8 Diaphragm	1/8 M. quadriceps
	1/8 M. quadriceps	1/8 M. gluteus maximus
Period until complaints normalized	<3 months (n = 2)	<3 months (n = 3)
	>3 months (n = 4)	>3 months (n = 4)
	2× missing information	1× missing information
Hospitalization	6 days (5–14)	7 days (5–14)
	2× not necessary	1× not necessary

* In two patients, HEV has been diagnosed prior to NA diagnosis. This time period is not included in the calculations.

**Table 2:** Affected side in HEV-infected patients with NA.

	Bilateral (8 Patients)	Unilateral Right (7 Patients)	Unilateral Left (1 Patient)
Age	47 years (41–57)	43 years (26–56)	48 years
Sex	6× male	5× male	1× male
	2× female	2× female	
Symptoms	7× pain + hypesthesia	5× pain + hypesthesia	pain + hypesthesia
	1× pain solely	2× pain solely	
Time to HEV diagnosis	45 days (1–270)	34 days (1–180) *	516 days before NA onset *
Positive diagnostic testing	4× serology	4× serology	serology + PCR
		2× serology + PCR	
	4× serology + PCR	1× PCR solely	
ALT (U/L)	535 (71–1331)	1031 (214–2366)	22
Performed diagnostic procedure			
EMG	5/8	6/7	1/1
NCS	6/8	4/7	1/1
MRI	4/8	5/7	1/1
Ultra-Sound	1/8	1/7	0/1
Period until complaints normalized	<3 months (n = 2)	<3 months (n = 2)	<3 months (n = 1)
	>3 months (n = 4)	>3 months (n = 4)	
	2× missing information	1× missing information	
Hospitalization	7 days (5–14)	7 days (5–14)	5 days
	1× not necessary	2× not necessary	

* In two patients, HEV has been diagnosed prior to NA diagnosis. This time period is not included in the calculation.

**Table 3:** Drug therapy.

	Specific Therapy (8 Patients)	Non-Specific Therapy (8 Patients)
Glucocorticoids	8/8	0/8
Ribavirin	3/8	0/8
Immunoglobulins	1/8	0/8
Pregabalin	1/8	5/8
No drugs	0/8	3/8
Age	50 years (47–57)	41 years (26–47)
Sex	6× male	6× male
	2× female	2× female
Symptoms	7× pain + hypesthesia	6× pain + hypesthesia
	1× pain solely	2× pain solely
Time to HEV diagnosis	39 days (1–180) *	41 days (0–270) *
Positive diagnostic testing	4× serology	4× serology
	3× serology + PCR	4× serology + PCR
	1× PCR solely	
ALT (U/L)	655 (22–2366)	821 (214–1840)
Period until complaints normalized	<3 months (n = 3)	<3 months (n = 2)
	>3 months (n = 3)	>3 months (n = 5)
	2× missing information	1× missing information
Hospitalization	<7 days (n = 4)	<7 days (n = 4)
	>7 days (n = 4)	>7 days (n = 1)
		No hospitalization (n = 3)
	Mean: 8 days (5–14)	Mean: 4 days (0–11)

* In two patients, HEV has been diagnosed prior to NA diagnosis. This time period is not included in the calculations.

**Table 4:** Hospitalization time.

	No Hospitalization Necessary (3 Patients)	Hospitalization < 7 Days (8 Patients)	Hospitalization > 7 Days (5 Patients)
Age	40 years (32–46)	45 years (26–48)	52 years
Sex	2× male	6× male	4× male
	1× female	2× female	1× female
Noticeable pre-existing conditions	None (n = 3)	Heart transplant (n = 1) Herniated disc (n = 1) Arterial hypertension (n = 1) Bronchial asthma (n = 1)	Multiple sclerosis (n = 1) Multiple intervertebral disc protrusion (n = 1)
Symptoms	2× pain + hypesthesia	7× pain + hypesthesia	4× pain + hypesthesia
	1× pain solely	1× pain solely	1× pain solely
Time to HEV diagnosis	7 days (1–14) *	10 days (0–51) *	97 days (8–270)
Affected side	2/3 unilateral right 1/3 bilateral	3/8 unilateral right 1/8 unilateral left 4/4 bilateral	2/5 unilateral right 3/5 bilateral
ALT (U/L)	622 (335–900)	830 (22–2366)	641 (99–1330)
Affected muscles	3/3 M. serratus anterior 2/3 M. deltoideus 2/3 M supraspinatus 2/3 M. infraspinatus 1/3 M. quadriceps 1/3 M. gluteus maximus	6/8 M. serratus anterior 6/8 M. deltoideus 5/8 M. supraspinatus 5/8 M. infraspinatus	3/5 M. serratus anterior 3/5 M. deltoideus 2/5 M supraspinatus 2/5 M. infraspinatus 2/5 Diaphragm 1/5 M. quadriceps 2× missing information
Period until complaints normalized	>3 months (n = 2)	<3 months (n = 4)	<3 months (n = 1)
		>3 months (n = 3)	>3 months (n = 3)
	1× missing information	1× missing information	1× missing information
Hospitalization	None	6 days (5–7)	12 days (8–14)

* In two patients, HEV has been diagnosed prior to NA diagnosis. This time period is not included in the calculations.
